# Quality of early childhood education and care in Japanese accredited nursery centers: A study using the Early Childhood Environment Rating Scale, Third Edition (ECERS-3)

**DOI:** 10.1371/journal.pone.0281635

**Published:** 2023-02-10

**Authors:** Keiko K. Fujisawa, Taiyo Fukai, Makiko Nakamuro

**Affiliations:** 1 Department of Education, Faculty of Letters, Keio University, Tokyo, Japan; 2 Faculty of Humanities and Social Sciences, University of Tsukuba, Ibaraki, Japan; 3 Faculty of Policy Management, Keio University, Kanagawa, Japan; 4 The Tokyo Foundation for Policy Research, Tokyo, Japan; University of Sharjah, UNITED ARAB EMIRATES

## Abstract

This study presents the first quantitative evaluation of the quality of early childhood education and care (ECEC) in Japan to make a significant contribution to the body of knowledge accumulated on ECEC in countries where research has been limited. We observed 30 classes comprising 3-year-olds, 28 classes comprising 5-year-olds, and 30 classes comprising mixed-ages from publicly provided nursery centers under the jurisdiction of the Kanto metropolitan area, Japan. An internationally-recognized quality rating scale for ECEC called the Early Childhood Environment Rating Scale, 3rd edition, which consists of six subscales, was used for this study. In contrast to previous studies conducted in the US, the results of this study showed that the Japanese ECEC is characterized as showing higher scores in the two subscales, “Personal Care Routines” and “Interaction,” and showing lower score in the subscale, “Learning Activities.” In addition, this study showed that the quality of ECEC varied across nursery centers. Furthermore, with regard to the two subscales, “Interaction” and “Language and Literacy,” the degree of variation within centers differed across nursery centers. This study analyzed how these characteristics of Japanese ECEC can be partly produced by the existence of national guideline for nursery centers authorized by the Japanese government. In addition, mechanisms producing differences in the quality of ECEC among and within centers were also discussed.

## Introduction

Early childhood education and care (ECEC) has recently drawn growing attention in interdisciplinary fields of study, not only in pedagogy and developmental psychology, but also in economics and others. The interest is not limited to academia, and has become an extremely practical field of study, with increasing attention being paid to it in policy making. In particular, there is a growing emphasis on the contribution of a high-quality environment in childhood to the healthy development of children in later life. The pioneering the Perry Early Childhood Education Project was the first of its kind. Initiating in the US during the 1960s, the Perry Early Childhood Education Project aimed to provide an intensive ECEC program for African American children growing up in low-income families and living in poor neighborhoods. In the project, targeted children experienced 2.5 hours of high-quality preschool program on weekdays and weekly home visits by experienced teachers. This intervention was started when children were age 3 and was continued for 2 years. Researches on this program show broad, long-term positive effects of this program, including schooling, economic activity, and marital life on the targeted children; the effect is summarized to be as much as 7 to 10 percent of the annual social rate of return [1].

These findings have been further confirmed by more recent longitudinal studies, leading enhancement of the importance of ECEC experience and its quality. For example, the widely available ECEC programs were shown to have a positive impact on various aspects of development including language, cognitive, and academic skills, and social-emotional skills [2, 3]. Studies have shown that the magnitude of the effects induced by ECEC programs on children’s development decreases with each increasing year of schooling [2, 4]. However, utilizing ECEC programs has been shown to have a positive impact on reducing problematic behaviors during adolescence and enhancing social skills during adulthood. Thus, the long-term effects of ECEC programs have also been recognized [5, 6]. In addition, using ECEC programs has demonstrated increased levels of a strong protective effect among children growing up in socio-economically disadvantaged families [3, 4, 6–9]. Note that, this interaction was not found in a large-scale study conducted in the US [10].

In addition to contributing to the healthy and adaptive development of children, the ECEC programs have several other benefits. The impact of reducing the access age for ECEC programs and strengthening regulations on the quality of ECEC programs in contribution to educational reforms was studied and implemented in Spain. The study found that children who participated in ECEC programs experienced increased lifetime earnings as a result of improved cognitive skills. which, in turn, resulted in increased tax revenues [11]. The study also found that the cost-effectiveness of fiscal spending on ECEC can reach fourfold. Moreover, the rate of social return to investments in ECEC is higher in other age groups [12]. Thus it can be seen that the provision of ECEC has potential benefits that can be experienced by both the children and society as a whole. Therefore, achieving universal access to ECEC is an important policy issue not only for developing children but also for the current and future societies.

However, merely increasing the availability of ECEC facilities does not suffice. In the Canadian province of Quebec, the introduction of fixed fees and rapid expansion of childcare capacity resulted in many children being placed in poor-quality childcare facilities [13]. This implementation resulted in long-term negative effects on the children’s adaptive development from preschool throughout adolescence [14]. Other studies on early placement in poor-quality childcare facilities showed an increased negative impact on children’s adjustment [15]. In Germany, measures to make ECEC available to children aged three years and older for four hours per week have resulted in a gradual increase in the number of young children enrolled in childcare facilities. A study examining the effects of universal access to ECEC in Germany [8] found that children with working and well-educated parents tended to start using childcare facilities earlier in the program. However, the effects of accessing the ECEC on the children from these families were not substantial. In contrast, children from socio-economically disadvantaged families, such as immigrants, only began using the ECEC facilities at a later stage. However, the effect of experiencing the ECEC facilities was greater among these families. This is also reported in studies on the effectiveness of universal access to ECEC conducted in Norway [16] and Italy [17]. These findings suggest that children from socio-economically disadvantaged backgrounds have developmental disadvantages because they are less likely to experience a quality and nurturing environment at home. However, these developmental disadvantages can be mitigated through accessing ECEC. In contrast, children from socio-economically advantaged families have various resources to enhance their development, both internal and external to their home. The use of childcare facilities might impede the positive development of these children if the quality is not assured as using childcare facilities reduced the experience of those resources. Therefore, it is important to improve the quality of ECEC for it to be beneficial for the development of all children.

### Evidence requirements for the effectiveness of the quality of ECEC in Japan

In Japan, almost all children have utilized various types of ECEC facilities before entering elementary schools (73.6% at 3-years-old, 99.2% at 5-years-old) [18]. Therefore, the impact of ECEC is pertinent for almost all children in Japan.

Historically, ECEC in Japan has been characteristic of accepting children as they are, emphasizing children’s independent and cooperative activities through daily life and play in childcare. Furthermore, these ECECs underscore the importance of supporting both the children’s development as well as the families raising them [19]. Therefore, this unique approach to ECEC in Japan may have different effects on the development of both the children and society, as opposed to findings obtained in other countries.

In addition to the content, there are various aspects affecting ECEC in Japan, which differs from programs in other countries. There are three types of ECEC facilities in Japan that are under the jurisdiction of three different ministries, namely, kindergartens (Youchi-en), nursery centers (Hoiku-sho), and Centers for early education and care (Kodomo-en). Furthermore, each kindergarten is accredited by a local government under the authority of the Ministry of Education, Culture, Sports, Science and Technology, Japan, and is regulated by the School Education Law and the Course of study for Kindergarten (Ministry of Education, Culture, Sports, Science, and Technology, effective in 2018) [20]. By definition, kindergartens provide care for children aged between three and six years. Nursery centers provide care for children whose parents cannot look after them at home due to work and other reasons. Furthermore, nursery centers are authorized by the Ministry of Health, Labor and Welfare, Japan, and regulated by the Child Welfare Law and the Nursery Center Childcare Guidelines (Ministry of Health, Labour and Welfare, Japan, effective in 2018) [21]. Nursery centers usually provide care for children as young as zero years old. Nursery centers can be categorized as accredited nursery centers that meet legal standards or unaccredited nursery centers that meet certain legal standards but are not bound by various regulations. For nursery centers as well, the local government is responsible for accreditation. In addition, there are nursery centers designated only for children under the age of three. Kodomo-en have functions combining those of kindergartens and nursery centers and provide support for child-rearing families in local communities. Each Kodomo-en is accredited under the authority of the Cabinet Office, Japan, and is regulated by the Guidelines for Centers for Early Childhood Education and Care [22].

Notably, despite the different authorities and laws regulating kindergartens, nursery centers, and Kodomo-en, the recently revised Course of Study for Kindergarten, the Nursery Center Childcare Guidelines, and the Guidelines for Centers for Early Childhood Education and Care are fairly similar in their content. Furthermore, these guidelines share the “Desirable Growth Figure in the Final Stage of Early Childhood,” which depicts a specific image of a child with certain qualities and abilities upon entering elementary school, which are nurtured throughout ECEC.

There are multiple qualifications generated by the systems that childcare providers can obtain. The most common way to obtain a kindergarten teaching license, that is, a national certification, is to enroll in a university or a junior college and complete a kindergarten teaching course and graduate with a department of early childhood education. The most common route to obtain a nursery center teacher qualification, which is also a national certification, is to enroll in a university, junior college, or special training college and complete a nursery teacher training program or the prescribed specialized education. Individuals with an educational qualification from a junior college, special training college, or higher can obtain the qualification after passing the nursery center teacher examination. However, individuals with only a high school or junior high school background can qualify to be a nursery teacher after passing the national examination and gaining substantial working experience at a child welfare-related facility.

The type of facility, the qualification system, as well as the frequency of the ECEC experience among Japanese children varies from the experience of children living in other countries. In the US, infants and children up to 24 months experienced ECEC for approximately 24 hours and 32 hours per week, respectively [23, 24]. Generally, facility-based childcare does not have sufficient opening hours to accommodate the working hours of parents. As a result, parents often combine multiple childcare options, which include using nannies, as opposed to focusing on a single early childhood education facility [25]. Thus, when considering the effectiveness of ECEC, it is necessary to distinguish between the most effective childcare experiences.

On the contrary, standard nursery centers in Japan open 11 hours a day, with 90% of children spending more than seven hours a day there on an average of five to six days a week [3]. According to another survey [26], children spend 9.5 hours in nursery centers on weekdays. This means that children spend an average of 47.5 hours per week in nursery centers during weekdays alone. Similarly, while kindergartens in Japan are designed as half day care, many children spend an extended period in kindergarten. The regulating guidelines for Kindergarten [20]allocate four hours of “instructional time” per day, even though many kindergartens provide childcare beyond this recommended time. According to the Survey on the Actual Conditions of Preschool Education [27], 87.8% of kindergartens (public kindergartens: 70.5%; private kindergartens: 96.9%) provide childcare services beyond the “instructional hours” for a five day week. In addition, more than 70% of kindergartens end childcare services after 5:00 p.m. This reality represents another unique aspect of ECEC in Japan. Despite the long hours spent in the same facility, children do not necessarily stay with the same caregivers all the time, as the working hours of the caregivers do not accommodate the length of the children’s stay.

As described, the ECEC experience varies in many aspects from a Japanese context. Thus, caution should be exercised when generalizing findings from this study to different socio-economic contexts external to Japan [7]. Therefore, it is necessary to examine the quality and practicality of ECEC in terms of the current social, economic, and cultural context of Japan. However, except for a limited few [28], there are no large-scale longitudinal studies that focus on ECEC and child development in Japan.

The lack of data on ECEC in Japan is attributable to the fact that there are currently no scales that have been developed to quantitatively evaluate the quality of ECEC from a Japanese context. However, translated versions of quality assessment scales were developed in countries outside Japan. One of them is the Early Childhood Environment Rating Scale (3rd edition; ECERS-3) [29], which was developed in the US. The ECERS is based on cognitive and attachment theories of developmental psychology. Furthermore, the ECERS provides a comprehensive assessment of the quality of ECEC using several indicators, including “process quality” and “structural quality” [30]. The first edition of the ECERS was published in 1980, and it is currently on its third edition. In addition to the US, the ECERS has been used to survey and monitor ECEC in a variety of countries that have different social, cultural, and economical contexts, including Scandinavian, Asian, and African countries [31–34].

The developers of the ECERS emphasize the importance of ensuring children’s health and safety, fostering their social-emotional development through positive relationships between themselves and adults, and building environments to foster children’s learning through experiences that stimulate their curiosity. These are important elements of the ECEC constant across countries and cultures [35]. Furthermore, using an internationally-recognized quality evaluation scale such as ECERS to examine the quality of ECEC in Japan, enables same-level axis comparisons of ECEC with other countries. This comparison is pertinent for assessing the strengths and opportunities related to the improvement of ECEC in Japan.

### Aims of this study

In Japan today, the number of nursery centers and the number of children enrolled in them are rapidly increasing due to the increase in the number of dual-earner households, while the number of kindergartens is on a downward trend, with a gap of more than three times as large [36]. In addition, the number of Kodomo-en is much smaller than the number of nursery centers and kindergartens, since the Kodomo-en system only launched in 2015. Therefore, this study focused the quality of ECEC in nursery centers.

This study aimed to quantitatively assess the quality of ECEC among preschool classes from accredited nursery centers in the Kanto region in Japan using the ECERS-3. In addition, this study aimed to identify the strengths and challenges of ECEC programs in Japan by comparing data from the US.

## Materials and methods

### Participating centers

The research team conducted a comprehensive survey of accredited nursery centers in the Kanto metropolitan area, Japan. This paper reports the results of the surveys on preschoolers’ classes (classes for 3-years-old, 5-years-old, and mixed-age classes) between 2017 and 2019. The survey results from the accredited nursery centers that only provided infant classes were not reported in this study due to the different rating scales [37] for those classes.

It is of note that every accredited nursery center that provides full-day ECEC is governed by the same laws and regulations. The entity could be established and operated either by the local government or a private organization. Hence, there are three types of accredited nursery centers. First is Public-Public, which are centers administered and operated by a public organization. The second is Public-Private, which are centers administered by the public but operated by a private organization. The third is Private-Private, which are centers administrated and operated by private organizations.

In 2017, 12 out of 17 accredited nursery centers in the city participated in the present study. Three accredited nursery centers did not participate due to conflict with childcare training programs conducted by the local government; two did not participate because their consent to the survey was not obtained; and one was closed at the end of the fiscal year. In 2018, 14 out of the 16 accredited nursery centers in the city participated in the survey. Two centers did not participate due to conflict with childcare training programs conducted by the local government. In 2019, all of the 18 accredited nursery centers in the city participated in the survey. The survey year and the type of nursery centers are shown in Table 1. Furthermore, the number of classes is listed by age group in Table 2.

**Table 1:** The survey year and the type of accredited nursery centers.

Fiscal year	Public-Public	Public- Private	Private-Private	Total
2017	0	2	10	12
2018	0	2	12	14
2019	2	2	14	18

**Table 2:** The number of classes per age group.

Fiscal year	3-years-old	5-years-old	Mixed age	Total
2017	8	8	8	24
2018	9	9	10	28
2019	13	11	12	36

This study was approved by the Keio University SFC Ethics Committee for Experiments and Research on December 23, 2017 (Reception No. 173). The local government obtained informed consent for this study from nursery centers. The heads of the nursery centers explained this study to the childcare staff and parents. In addition, the local government informed parents of their center participating in the present study.

### Procedure

#### ECERS-3

In this study, an observational survey on ECEC was conducted using the ECERS-3 [29]. There are six subscales used in the ECERS-3, namely; “Space and Furnishings”, “Personal Care Routines”, “Language and Literacy”, “Learning Activities”, “Interaction”, and “Program Structure.” Each subscale has 4–11 items, such as “Indoor space” in the subscale of “Space and Furnishings” and “Becoming familiar with print” in the subscale of “Language and Literacy”, with 35 items in total. There are approximately 15 indicators in each item, with 461 indicators in total. Assessors scored each indicator using Yes/No. Based on the evaluation of the indicators included in each item, a score from 1 to 7 is calculated for each item. In this study, each subscale score was calculated as the average of each item included in the subscale. The total average score was calculated as an average of the subscale scores.

Each survey occurred during one school year from June to January. The Japanese school year begins in April and ends in March. Two or three of several trained assessors visited each center for observation at 9 am for three and a half hours. Unless there was any risk, the assessors did not interact with the children in the class during the observation nor did they assist the childcare and education in the class. Each assessor conducted the scoring independently, and any discrepancies in the evaluation were resolved through discussion after the observation. This evaluation used was the agreed-upon for the analysis.

#### Assessors

The assessors were qualified in at least one aspect related to child development and education, namely, a kindergarten teacher, nursery teacher, and licensed psychologist. Furthermore, each of them had at least a junior college degree. Each assessor received at least 16 hours of training in lecture style to understand the details of what is evaluated by the ECERS-3 and its evaluation criteria, and at least three sessions of training in which they actually visited nursery centers to conduct ECERS-3 evaluations. In addition, they received at least 16 hours of training in lecture and/or discussion style each year. Although each visit was not necessarily conducted by the same combination of assessors, the average agreement rate was high(Mean (SD) = 90.0 (7.7) %). The differences in ratings among the assessors were examined by regression analysis to confirm that the differences among the assessors were not statistically significant. Specifically, we regressed ECERS-3 scores on the assessor flags for which they were assigned and performed a test of no difference for all assessors (a joint test of the null hypothesis that the coefficients on each assessor flag are all zero). The results of the test showed a p-value of 0.173 for the estimation assuming homoskedasticity in variance and a p-value of 0.082 for the estimation assuming heteroskedasticity in variance. Note that in the estimation, we control for the year, center, and target class of the evaluation.

#### Feedback

Feedback was provided to the nursery centers of the scores for the six subscales in ECERS-3 using box plots to show the lowest and highest scores, as well as the 25th and 75th percentile scores for each subscale from the participating nursery centers. In addition, the results for nursery centers that participated in surveys more than once were presented to compare with previous results. After observations, each assessor provided an independent evaluation of the ECERS-3, writing freely about the “good points” and “points that can be improved.” These notes were presented to each nursery center with minimum modifications to protect the identity of the individual caregivers and children.

## Results and discussion

### Descriptive statistics

The total average score and the scores for each subscale are shown in Table 3. Item 27 was excluded from the analyses as none of the classes observed any activities relating to “Appropriate use of technology.” Similarly, the item “Whole-group activities for play and learning” (item 35) was not included in the analyses since this item was not necessarily observed among all the sample in this study.

**Table 3:** The mean total average score and mean scores of each subscale.

Year	Total average	Subscale 1	Subscale 2	Subscale 3	Subscale 4	Subscale 5	Subscale 6
2017	3.07 (0.65)	2.96 (0.63)	4.03 (1.19)	3.12 (0.92)	1.96 (0.61)	4.53 (1.33)	3.35 (1.50)
2018	3.35 (0.57)	3.42 (0.64)	4.29 (1.20)	3.43 (0.82)	2.14 (0.61)	4.55 (1.38)	4.07 (1.37)
2019	3.68 (0.72)	3.94 (0.92)	4.50 (1.24)	3.47 (0.99)	2.43 (0.73)	5.08 (1.01)	4.40 (1.34)
Entire period	3.41 (0.69)	3.50 (0.86)	4.30 (1.21)	3.36 (0.92)	2.21 (0.68)	4.76 (1.24)	4.01 (1.44)

Note. Standard deviations are shown in parentheses. Definitions of subscales. Subscale 1: Space and Furnishing. Subscale 2: Personal Care and Routines. Subscale 3: Language and Literacy. Subscale 4: Learning Activities. Subscale 5: Interaction. Subscale 6: Program Structure.

The total average scores were not statistically different among the class age groups. Using the Tukey-Kramer test, it was found that the difference between the classes for three-year-olds and that for five-year-olds was 0.332 (SE = 0.179), with a 95% confidence interval (CI): [-0.095, 0.759]. Furthermore, the difference between the classes for the mixed-age group and three-year-olds was -0.033 (SE = 0.176), with a 95%CI: [-0.452, 0.387]. Finally, the difference between the classes for the mixed-age group and the one for five-year-olds was -0.364 (SE = 0.179), with a 95%CI: [-0.791, 0.063]). Based on these results, the following analyses conducted in this study were performed by undistinguishing the class age groups. The total average score and the scores for each subscale per class age group are shown in Table 4.

**Table 4:** The mean total average score and mean scores of total average and each subscale per class age group.

Class type	Total average	Subscale 1	Subscale 2	Subscale 3	Subscale 4	Subscale 5	Subscale 6
3-years-old	3.31 (0.70)	3.38 (0.84)	4.46 (1.27)	3.12 (0.81)	2.01 (0.60)	4.83 (1.15)	4.02 (1.39)
5-years-old	3.64 (0.70)	3.73 (0.96)	4.51 (1.14)	3.82 (1.00)	2.36 (0.72)	5.04 (1.04)	4.20 (1.48)
Mixed age	3.28 (0.64)	3.42 (0.75)	3.96 (1.19)	3.17 (0.81)	2.27 (0.70)	4.44 (1.46)	3.83 (1.48)

Note. Standard deviations are shown in parentheses. Definitions of subscales. Subscale 1: Space and Furnishing. Subscale 2: Personal Care and Routines. Subscale 3: Language and Literacy. Subscale 4: Learning Activities. Subscale 5: Interaction. Subscale 6: Program Structure.

The internal consistencies of each subscale ranged from good to modest: *α* = 0.57 for “Space and Furnishings”; *α* = 0.42 for “Personal Care Routines”; *α* = 0.58 for “Language and Literacy”; *α* = 0.72 for “Learning Activities”; *α* = 0.78 for “Interaction”; *α* = 0.52 for “Program Structure”. The internal consistency of the total average score was good (*α* = 0.87).

The subscale scores had a significant positive correlation with each other, except for the correlation between “Personal Care Routines” and “Learning Activities” and between “Personal Care Routines” and “Program Structure.” The excluded correlations were positive, however, they were not statistically significant (see Table 5). This suggests that while some classes generally scored high on various items, others had an overall low score.

**Table 5:** Correlations among the subscale scores and the total average.

	1	2	3	4	5	6	7
1 Space and Furnishings	—						
2 Personal Care Routines	0.368***	—					
3 Language and Literacy	0.458***	0.376***	—				
4 Learning Activities	0.609***	0.166	0.540***	—			
5 Interaction	0.479***	0.321**	0.523***	0.377***	—		
6 Program Structure	0.420***	0.294	0.479***	0.536***	0.521***	—	
7 Total average	0.797***	0.558***	0.764***	0.771***	0.745***	0.695***	—

Note. The statistical significance of correlation coefficients was adjusted with Bonferroni correction.

**: *p* <.05.

***: *p* <.01.

### Comparison with previous US studies

ECERS was developed in the United States and is now being used in a variety of countries, care must be taken when using it and interpreting scoring results in different cultural contexts because different cultural backgrounds in various countries would influence ECEC practices and goals in each country. By comparing data from previous studies in the U.S. with data from Japan, this paper does not simply look at which country’s ECEC is of higher quality, but would discuss what strengths and challenges exist in Japanese childcare practices, and how differences in cultural backgrounds between the U.S. and Japan affect childcare practices and goals, which are reflected in scores.

Since the ECERS-3 was only published in 2015, published studies on available item-level scores were limited when this study was conducted. Thus, Early et al. [38] and Montes et al. [39] were used for comparison with the data in the present study. Both studies aimed to confirm the factor structure of ECERS-3. However, previous studies differed significantly from the present study in terms of sample representation and sample sizes. Early et al. [38] was based on the data from 1,063 classes in three states in the US (Georgia, Pennsylvania, and Washington). While the Montes et al. [39] study was based on the data from 148 classes in a community in Rochester, New York, where ECERS has been used for training among directors and caregivers for over 20 years.

The total average scores and the subscale scores are shown in Table 6. The information on correlations between items was not available in the studies by Early et al. [38] and Montes et al. [39]. Thus, a statistical comparison was performed in this study for the item level, although it was not possible to statistically compare from the subscale level.

**Table 6:** Comparison of ECERS-3 subscales among the three studies.

	The present study	Early et al.	Montes et al.
Total average	3.41	3.64	5.20
Space and Furnishings	3.50	3.62	4.71
Personal Care Routines	4.30	3.36	4.84
Language and Literacy	3.36	3.54	5.24
Learning Activities	2.21	2.96	4.42
Interaction	4.76	4.41	6.10
Program Structure	4.01	3.98	5.87

Note. All data from 2017 to 2019 were included in the present study. Scores of Early et al. were calculated by the authors from Table 3 in Early, et al. [38]. Scores for the Montes et al. study were calculated by the authors using Table 1 in Montes, et al. [39].

As shown in Table 7, almost all item scores of the present study were significantly lower than those of Montes et al. [39]. However, comparisons with Early et al. [38], which was based on a more representative sample than Montes et al. [39], showed a different perspective. The item scores related to gross motor activities (“Space for gross motor play” (item 6), “Gross motor equipment” (item 7), and “Supervision of gross motor” (item 28) were significantly higher in the present study than those in Early et al. [38]. In addition, the items included in the “Personal Care Routines” subscale were all significantly higher in the present study than those in Early et al. [38], except for “Safety practices” (item 11), which indicated that there is no statistical difference. Furthermore, two of the five items included in the “Interaction” subscale were significantly higher in the present study than those of Early et al. [38]. The scores of the two items were higher in the present study than in Early et al. [38] although the differences were not statistically significant. The only exception was “Individualized teaching and learning” (item 29), with a present study score that was lower than the one of Early et al. [38]. Almost all of the items included in the “Learning activities” subscale were significantly lower in the present study than those of Early et al. [38]. This excludes the “Nature/science” (item 22) and “Math in daily events” (item 24) whose differences were not statistically significant.

**Table 7:** Comparison of the ECERS-3 item scores among the three studies.

	(1) Present study			(2) Early et al.			(3) Montes et al.			Test of differences (t-value)			
	N	Mean	SD	N	Mean	SD	N	Mean	SD	(1)—(2)		(1)—(3)	
Space and Furnishings													
1. Indoor space	88	6.03	1.06	1063	4.55	1.55	148	5.70	1.47	1.48	(8.81)	0.33	(1.87)
2. Furnishings for care, play, and learning	88	2.45	1.54	1063	4.05	1.10	148	4.97	1.28	-1.60	(-12.62)	-2.52	(-13.52)
3. Room arrangement for play and learning	88	2.63	1.20	1063	3.42	1.45	148	4.47	1.83	-0.80	(-5.00)	-1.85	(-8.44)
4. Space for privacy	88	2.75	1.27	1063	4.07	1.60	148	5.32	1.77	-1.32	(-7.54)	-2.57	(-11.92)
5. Child-related display	88	2.64	1.80	1063	3.24	1.37	148	4.84	1.88	-0.60	(-3.87)	-2.20	(-8.84)
6. Space for gross motor play	88	4.40	1.98	1063	3.18	1.42	148	3.68	1.73	1.22	(7.47)	0.72	(2.92)
u. Gross motor equipment	88	3.64	2.16	1063	2.80	1.68	148	4.02	2.27	0.84	(4.38)	-0.38	(-1.28)
Personal Care Routines													
8. Meals/Snacks	88	4.42	1.46	1063	3.15	1.29	148	4.70	1.79	1.27	(8.78)	-0.28	(-1.24)
9. Toileting/diapering	88	4.32	2.07	1063	3.21	1.41	148	4.42	2.11	1.11	(6.79)	-0.10	(-0.36)
10. Health practices	88	4.10	1.90	1063	3.06	1.40	148	4.75	1.99	1.04	(6.51)	-0.65	(-2.46)
11. Safety practices	88	4.38	2.48	1063	4.03	1.72	148	5.48	1.85	0.35	(1.74)	-1.11	(-3.90)
Language and Literacy													
12. Helping children expanding vocabulary	88	3.05	1.41	1063	3.24	1.42	148	5.49	1.66	-0.19	(-1.24)	-2.44	(-11.55)
13. Encouraging children to use language	88	3.80	1.65	1063	4.20	1.54	148	6.07	1.51	-0.40	(-2.36)	-2.27	(-10.81)
14. Staff use of books with children	88	3.63	1.84	1063	3.38	1.69	148	4.97	1.91	0.25	(1.30)	-1.35	(-5.30)
15. Encouraging children to use books	88	3.48	1.20	1063	3.69	1.47	148	4.89	1.55	-0.21	(-1.32)	-1.41	(-7.34)
16. Becoming familiar with print	88	2.86	1.37	1063	3.19	1.24	148	4.76	1.49	-0.33	(-2.35)	-1.90	(-9.75)
Learning Activities													
17. Fine motor	88	3.11	1.70	1063	3.98	1.59	148	5.55	1.59	-0.87	(-4.88)	-2.44	(-11.08)
18. Art	88	2.95	1.78	1063	3.43	1.48	148	5.09	1.67	-0.48	(-2.85)	-2.14	(-9.27)
19. Music and movement	88	2.35	1.25	1063	3.15	1.17	148	4.43	1.42	-0.80	(-6.11)	-2.08	(-11.35)
20. Blocks	88	1.47	0.83	1063	2.23	1.26	148	3.59	1.51	-0.76	(-5.59)	-2.12	(-12.15)
21. Dramatic play	88	2.16	1.39	1063	3.14	1.66	148	4.69	1.93	-0.98	(-5.39)	-2.53	(-10.76)
22. Nature/science	88	2.35	1.37	1063	2.54	1.17	148	4.07	1.72	-0.19	(-1.43)	-1.72	(-7.98)
23. Math materials and activities	88	1.41	0.89	1063	2.29	1.34	148	4.11	1.85	-0.88	(-6.06)	-2.70	(-12.84)
24. Math in daily events	88	3.14	1.53	1063	2.99	1.43	148	4.81	1.76	0.15	(0.92)	-1.67	(-7.41)
25. Understanding written numbers	88	1.44	0.77	1063	1.73	1.15	148	3.01	2.05	-0.29	(-2.30)	-1.57	(-6.89)
26. Promoting acceptance of diversity	88	1.70	0.75	1063	4.07	1.19	148	4.82	1.34	-2.37	(-18.35)	-3.12	(-20.04)
27. Appropriate use of technology	-	-	-	291	3.14	1.86	-	-	-	-	-	-	-
Interaction													
28. Supervision of gross motor	84	5.08	1.76	1063	4.11	1.74	148	5.47	2.16	0.97	(4.93)	-0.39	(-1.40)
29. Individualized teaching and learning	88	3.95	1.78	1063	4.32	1.70	148	6.36	1.45	-0.37	(-1.93)	-2.41	(-11.30)
30. Staff-child interaction	88	5.24	1.68	1063	4.97	1.84	148	6.47	1.27	0.27	(1.32)	-1.23	(-6.36)
31. Peer interaction	88	4.75	1.54	1063	4.47	1.56	148	6.12	1.28	0.28	(1.62)	-1.37	(-7.36)
32. Discipline	87	4.82	1.60	1063	4.18	1.42	148	6.08	1.54	0.64	(3.98)	-1.26	(-5.98)
Program Structure													
33. Transitions and waiting times	88	4.30	1.76	1063	3.90	1.92	148	5.76	2.00	0.40	(1.87)	-1.46	(-5.68)
34. Free play	88	3.73	1.75	1063	4.06	1.51	148	5.98	1.50	-0.33	(-1.96)	-2.25	(-10.48)
35. Whole-group activities for play and learning	76	4.84	1.77	1044	3.80	1.50	-	-	-	-	-	-	-

Note. Data for the present study includes all data from 2017 to 2019. Scores of Early et al. were calculated by the authors using Table 3 from Early et al. [38]. Scores of Montes et al. were calculated by the authors using Table 1 from Montes et al. [39].

The Nursery Center Childcare Guidelines [21], which regulate ECEC in nursery centers in Japan, state that caregivers should provide a clean and safe environment; satisfy children’s physiological needs through appropriate assistance and responsive interaction with children; and appropriately assist children so that they are motivated in activities such as eating, toileting, dressing/undressing, and personal cleanliness. These are related to the contents included in the “Personal Care Routine” subscale in ECERS-3. Furthermore, the guideline states that caregivers should accurately understand the condition and developmental process of each child, interact with the child in a responsive manner that addresses the child’s needs appropriately. Moreover, caregivers ought to build a relationship of trust with each child by accepting and empathizing with their feelings. Based on a relationship of trust with themselves, caregivers should watch over the process of children’s growth and promote their development for children to engage actively and enhance their self-motivation and willingness to explore. These aspects relate clearly to the contents included in the “Interaction” subscale in ECERS-3. The findings from this study showed that the scores of the items included in the “Personal Care Routine” and “Interaction” subscales in ECERS-3 were generally higher, which suggests that ECEC provided at accredited nursery centers aligns well with the general framework of the Nursery Center Childcare Guidelines regulates. Furthermore, this indicates good quality compared to the childcare facilities in the US. Thus, it could be said that ECEC in Japan is robust in terms of the aspects related to “Personal Care Routines” and “Interaction.” However, most scores were significantly lower than preschools receiving increased intensive interventions using ECERS [39]. This would mean that there is still room for improvement in the quality of ECEC in Japan.

The present study found that only the “Individualized teaching and learning” item from the “Interaction” subscale in ECERS-3 was in a lower direction than in either of the two previous U.S. studies. This difference can be understood as the difference between American ECEC emphasis on individualism and avoidance of whole-group activities due to negative feelings toward groupism, and Japanese ECEC emphasis on relationships with peers, learning as a whole group, and doing things for oneself in a group setting [40]. This orientation of Japanese early education teachers is reflected, for example, in the practice that when a conflict between children, the teacher does not immediately get involved with the parties involved individually and mediate the conflict, but encourages the group, including the children who are not involved in the conflict, to work together to find a solution to the conflict. They believe that as well as children learning to resolve conflicts and learn prosocial behavior through their individual experiences, the group of children can regulate the social behavior of the children, which teachers should support them to do so [41]. This is reflected in the regulation of the Nursery Center Childcare Guidelines. Similar to ECERS emphasizes the importance of providing individualized instructions that align with the child’s developmental process and interests [29], this is emphasized in the Nursery Center Childcare Guidelines. At the same time, the guideline states for children aged three years and above that “In the childcare of children at this age, group activities as well as individual child’s growth should be enriched” in the basic aims and contents of ECEC [21]. Furthermore, it states that children should be able to express themselves and act with confidence in group life contexts and that they should develop a sense of normality and the ability to regulate their own feelings through experiencing group life in nursery centers [21]. Thus experiences and learning in group activities and group life are emphasized in the context of ECEC in Japan, which might make more difficult for caregivers to balance individualized involvement with each child and to provide group activities for children.

The item scores for the “Learning Activities” subscale in this study were particularly low in comparison to the previous two U.S studies. To meet with criteria of ECERS-3, it is necessary to provide play materials and equipment rich in both quality and quantity for the wide range of learning activities. The mere presence of equipment and materials in classrooms is not considered an achievement in terms of the indicators in ECERS-3. However, the environment must be accommodating so that children can play freely and choose to use the equipment and materials. These detailed criteria are not described in the Nursery Center Childcare Guidelines [21]. This may reflect that there is an important difference between the American early education teachers emphasis on individual children choosing during “activity center time” which among a handful of activities to pursue, while the Japanese early education teachers emphasis on children choosing what to play with less constraint on the options, less specific learning goals, and less structuring of the activities by teachers [40] (Tobin, Hsueh, & Karasawa, 2009). It is observed that, in contrast to the US teachers presenting children with a list of options to choosing from play materials, Japanese children are free to roam the classrooms and grounds, choosing activities without teachers’ presenting or defining options [40] (Tobin et al., 2009). In addition, it is important to consider the amount of time that can be allocated and available for free play. In Japan’s ECEC context, free play as well as group activities are emphasized, which may impact daily routines, thus making it difficult to meet the required criteria for free play as outlined in the ECERS-3 scoring. However, it is important to note that this issue is not unique to the Japanese childcare context since Early, et al. [38] also found that the “Learning Activities” subscale scores were relatively lower than the scores reported by the other subscales, as observed in this study.

### Differences among accredited nursery centers

**Fig 1:**
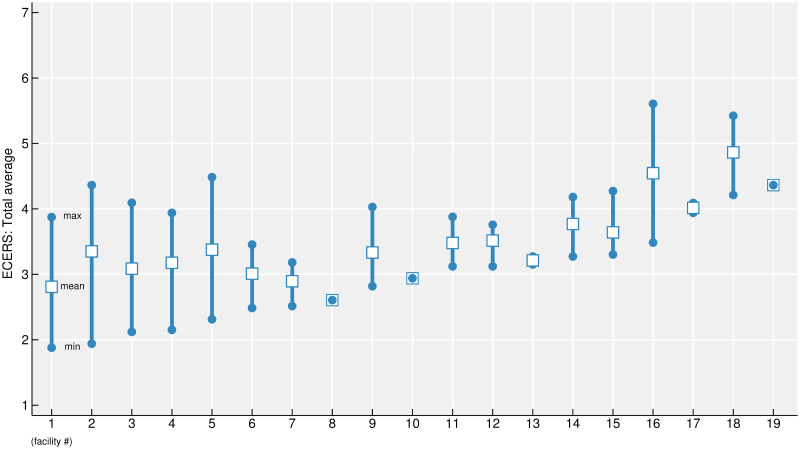
ECERS total average scores for each facility. Fig 1 shows the lowest and highest scores for each facility. These findings are important to visually examine the differences in scores within a facility.

To determine whether differences were present in the quality of ECEC among nursery centers, Fig 1 illustrates the mean of the total average score for each facility. Furthermore, it shows the lowest and highest scores for each facility. These are important to visually examine the differences in scores within a facility. Fig 1 illustrates that the mean of the total average score of each facility varies among centers. In addition, the lowest and highest scores differ vastly in some facilities, while in other facilities, the differences in scores are relatively small.

The variations among the total average score and each subscale score between nursery centers were examined using ANOVA, where the variations between the facilities and between survey years were taken into account. As shown in Table 8, the total variance was 42.85% of the total average score, and 31.46% to 44.98% of the other subscales were explained by the differences between the facilities. These between-facility differences were statistically significant, suggesting that there were major differences in the quality of ECEC provided by the nursery centers. Furthermore, the heterogeneity of variance among the facilities was examined using Breusch–Pagan test. The findings showed that the variation of scores in the “Language and Literacy” and “Interaction” subscales varied among facilities (see Table 8). The findings underscored the heterogeneity of the variance of these subscales within the facilities, where some facilities had small variations in scores within classes, while others had scores that varied widely within classes.

**Table 8:** Results of ANOVA for testing the score variations between nursery centers.

	Total average	Subscale 1	Subscale 2	Subscale 3	Subscale 4	Subscale 5	Subscale 6
%Explained	43.40%	42.85%	35.65%	37.38%	44.98%	31.96%	43.03%
F-value	3.70***	4.55***	2.14**	2.32***	3.59***	1.84**	3.33***
BP-test (chi2)	0.44	0.62	1.95	3.53*	1.01	12.15***	0.06

Note. N = 88. Definitions of subscales. Subscale 1: Space and Furnishing. Subscale 2: Personal Care and Routines. Subscale 3: Language and Literacy. Subscale 4: Learning Activities. Subscale 5: Interaction. Subscale 6: Program Structure. In the calculation of the score for “Learning Activities”, the item “Appropriate use of technology” (item 27) was not included. In the calculation of “Program Structure”, item 35 “Whole-group activities for play and learning” was not included. The variations between facilities and between survey years were taken into account in ANOVA. %Explained: percentage of the total variation explained by the variation between facilities. BP-test (chi2): test statistics of Breusch-Pagan test.

*: *p* <.10.

**: *p* <.05.

***: *p* <.01.

In Japan, nursery centers that meet certain standards can be approved as accredited nursery centers. The Standards on Equipment and Operation of Child Welfare Institutions [42],detail the provisions for the requirements on accredited nursery centers related to “structure quality” such as staffing and facilities. However, provisions on “process quality” have not been outlined adequately despite their direct association with child development. This might be related to the results of this study indicating that not all accredited nursery centers provide the same level of quality ECEC. In Japan, the local government determines childcare fees paid by parents to accredited nursery centers. This calculation is based on the parents’ income and other factors. However, the fees are not influenced by the quality of ECEC provided by the facilities. The present findings suggest that the same amount of money paid by parents for childcare fees can not gurantee that their children experience the same level of quality of ECEC even at accredited nursery centers.

In a study of a representative sample in the US [43], it was reported that the quality of childcare varies widely across providers. The results showed differences in the children’s reading and arithmetic skills at age five, which varied depending on the providers, i.e., informal and formal childcare. This could explain the differences in the quality of ECEC among these providers. Formal childcare, such as Head Start and prekindergarten, is strictly regulated and maintains better quality regulation as opposed to informal childcare. However, when considering only the formal childcare providers, the quality dispersion was found to be relatively small, and the difference in children’s skills depending on providers is not explained by the difference in ECEC quality. Instead, the difference is explained by the differences in the children’s family backgrounds [43]. Thus, the difference in ECEC quality among the facilities found in this study may be relatively small since this study was limited to accredited nursery centers in one municipality. It is possible that the quality dispersion among different types of providers, such as unaccredited nursery centers and kindergartens, would be larger than the one found in the present study.

The findings here also reveal differences among nursery centers in terms of the differences within the facilities. In the “Language and Literacy” and “Interaction” subscales, the variation in scores within facilities differed across nursery centers (see Table 8). The “Interaction” and “Language and Literacy” subscales place increased focus on the interaction and care between caregivers and children, as opposed to physical environment elements such as play equipment and teaching materials. Therefore, these subscales may provide a clear picture of the quality provided by caregivers that is not easily reflected in the physical environment. Thus, this finding suggests that the quality created by caregivers varies even within one center and the magunitude of the variation within one center varies across nursery centers.

The mechanisms of this finding possibly result from the differences in leadership among facility directors and head caregivers. The leaders of facility directors influence a wide range of aspects that directly or indirectly contribute to the quality of ECEC provided by caregivers [44, 45]. For example, depending on the leadership style, there would be varying degrees of sharing of views on children, childcare, and the philosophy and educational policy of the nursery center among caregivers. Furthermore, the degree of independence and autonomy that each caregiver can exercise in their daily practices would differ. In addition, the leadership of the facility directors will also be responsible for responding to the needs of individual caregivers, such as ensuring a work-life balance for caregivers and guaranteeing training opportunities that improve their professional development as caregivers. The demonstration of daily practices among caregivers in nursery centers differs depending on the leadership style demonstrated by the directors. However, it is beyond the scope of this study to demonstrate this dynamic. Future research would therefore be necessary to examine the mechanism, where factors such as leadership can vary among nursery centers to influence the daily practice of caregivers. Furthermore, to analyze the difference among caregivers within a center as this would emerge differently among different nursery centers.

## Limitations of this study

First, it should be noted that the observations of the present study were conducted at accredited nursery centers under the jurisdiction of one municipality in the Kanto region, Japan. Thus, the study does not reflect a representative sample of ECEC facilities in Japan, which, instead, comprises multiple types of facilities such as kindergartens and unaccredited nursery centers. Second, the sample size of the present study was not large. Hence, it is important to accumulate general data on ECEC in Japan for a larger sample that includes a variety of facility types. Third, the internal consistencies of the subscales were modest, despite a good score for internal consistency of the total average. However, this may be due to the nature of the ECERS-3 scale structure, as opposed to the data or sample used in this study. For example, “Meals/snacks” (item 8) includes indicators for hygiene and social interaction. Despite being included in the “Personal care routine” subscale, the aspect of interactions among caregivers and children at mealtime can also be included in the “Interaction” subscale. It is pointed that there are more than 60 indicators that can be included in the “Interaction” subscale which are included in subscales other than the “Interaction” subscale [46]. Thus, the fact that a single item contains multiple aspects of ECEC adds to the difficulty of ensuring internal consistency among the assessment contents of each item. This can also be attributed to the six subscales presented by ECERS-3 yet are not expressed as a data-based factor structure [38]. It can therefore be concluded that the psychometric challenges are inherent to the ECERS-3. Finally, this study demonstrated the differences in childcare quality among accredited nursery centers operating under the same standards, however, it was beyond the scope of this study to examine the mechanisms underlying these differences. It would be possible that family characteristics and other factors such as director’s leadership and management affected ECERS scores. Future research is necessary to explore the determinants of childcare quality using more detailed data such as family, children and teachers.

## Conclusion

This study is the first to provide a descriptive analysis of quantitative assessments of the quality of ECEC in Japan, using the internationally-recognized quality rating scale for ECEC, the ECRS-3. Based on the comparison to previous studies conducted in the U.S., this study showed that the Japanese ECEC is robust in aspects related to “Personal Care Routines” and “Interaction” in ECERS-3, and faces challenges in the aspect related to “Learning Activities” in ECERS-3. These findings may be related to the Nursery Center Childcare Guidelines governing Japanese nursery centers adhere. The study also showed that the scores varied between nursery centers despite the lack of diversity among the sample of accredited nursery centers within the jurisdiction of the municipality. Furthermore, the variations in scores for the “Interaction” and “Language and Literacy” within the centers differed across the centers. These findings suggest future research could implement a mechanism where varying factors among nursery centers can account for the difference among centers as well as the difference within the centers.

## Supporting information

S1 File Tables and details of summary statistics of ECERS-3. (DOCX)
