# Supplementary material for: Quality of early childhood education and care in Japanese accredited nursery centers: A study using the Early Childhood Environment Rating Scale, Third Edition (ECERS-3)
Source: PLoS One. 2023 Feb 10;18(2):e0281635. doi: 10.1371/journal.pone.0281635 (PMC9916609; doi:10.1371/journal.pone.0281635)
Supplement: S1 File — (DOCX) [file pone.0281635.s001.docx]

| **Table 1.** The survey year and the type of accredited nursery centers | | | | |  |
| --- | --- | --- | --- | --- | --- |
|  | Fiscal year | Public-Public | Public- Private | Private-Private | Total |
|  | 2017 | 0 | 2 | 10 | 12 |
|  | 2018 | 0 | 2 | 12 | 14 |
|  | 2019 | 2 | 2 | 14 | 18 |

| **Table 2.** The number of classes per age group | | | |  |  |
| --- | --- | --- | --- | --- | --- |
|  | Fiscal year | 3-years-old | 5 -years-old | Mixed age | Total |
|  | 2017 | 8 | 8 | 8 | 24 |
|  | 2018 | 9 | 9 | 10 | 28 |
|  | 2019 | 13 | 11 | 12 | 36 |

| **Table 3.** The mean total average score and mean scores of each subscale | | | | |  |  |  |  |
| --- | --- | --- | --- | --- | --- | --- | --- | --- |
|  | Year | Total average | Subscale 1 | Subscale 2 | Subscale 3 | Subscale 4 | Subscale 5 | Subscale 6 |
|  | 2017 | 3.07 (0.65) | 2.96 (0.63) | 4.03 (1.19) | 3.12 (0.92) | 1.96 (0.61) | 4.53 (1.33) | 3.35 (1.50) |
|  | 2018 | 3.35 (0.57) | 3.42 (0.64) | 4.29 (1.20) | 3.43 (0.82) | 2.14 (0.61) | 4.55 (1.38) | 4.07 (1.37) |
|  | 2019 | 3.68 (0.72) | 3.94 (0.92) | 4.50 (1.24) | 3.47 (0.99) | 2.43 (0.73) | 5.08 (1.01) | 4.40 (1.34) |
|  | Entire period | 3.41 (0.69) | 3.50 (0.86) | 4.30 (1.21) | 3.36 (0.92) | 2.21 (0.68) | 4.76 (1.24) | 4.01 (1.44) |
| Note. Standard deviations are shown in parentheses. Definitions of subscales. Subscale 1: Space and Furnishing. Subscale 2: Personal Care and Routines. Subscale 3: Language and Literacy. Subscale 4: Learning Activities. Subscale 5: Interaction. Subscale 6: Program Structure. | | | | | | | | |

| **Table 4.** The mean total average score and mean scores of total average and each subscale per class age group | | | | | | |  |  |
| --- | --- | --- | --- | --- | --- | --- | --- | --- |
|  | Class type | Total average | Subscale 1 | Subscale 2 | Subscale 3 | Subscale 4 | Subscale 5 | Subscale 6 |
|  | 3-years-old | 3.31 (0.70) | 3.38 (0.84) | 4.46 (1.27) | 3.12 (0.81) | 2.01 (0.60) | 4.83 (1.15) | 4.02 (1.39) |
|  | 5-years-old | 3.64 (0.70) | 3.73 (0.96) | 4.51 (1.14) | 3.82 (1.00) | 2.36 (0.72) | 5.04 (1.04) | 4.20 (1.48) |
|  | Mixed age | 3.28 (0.64) | 3.42 (0.75) | 3.96 (1.19) | 3.17 (0.81) | 2.27 (0.70) | 4.44 (1.46) | 3.83 (1.48) |
| Note. Standard deviations are shown in parentheses. Definitions of subscales. Subscale 1: Space and Furnishing. Subscale 2: Personal Care and Routines. Subscale 3: Language and Literacy. Subscale 4: Learning Activities. Subscale 5: Interaction. Subscale 6: Program Structure. | | | | | | | | |

| **Table 5.** Correlations among the subscale scores and the total average | | | | |  |  |  |  |
| --- | --- | --- | --- | --- | --- | --- | --- | --- |
|  |  | 1 | 2 | 3 | 4 | 5 | 6 | 7 |
|  | 1 Space and Furnishings | — |  |  |  |  |  |  |
|  | 2 Personal Care Routines | 0.368*** | — |  |  |  |  |  |
|  | 3 Language and Literacy | 0.458*** | 0.376*** | — |  |  |  |  |
|  | 4 Learning Activities | 0.609*** | 0.166 | 0.540*** | — |  |  |  |
|  | 5 Interaction | 0.479*** | 0.321** | 0.523*** | 0.377*** | — |  |  |
|  | 6 Program Structure | 0.420*** | 0.294 | 0.479*** | 0.536*** | 0.521*** | — |  |
|  | 7 Total average | 0.797*** | 0.558*** | 0.764*** | 0.771*** | 0.745*** | 0.695*** | — |
| Note. The statistical significance of correlation coefficients was adjusted with Bonferroni correction. ** : *p < .*05. *** : *p < .*01. | | | | | | | | |

| **Table 6.** Comparison of ECERS-3 subscales among the three studies | | | |  |
| --- | --- | --- | --- | --- |
|  |  | The present study | Early et al. | Montes et al. |
|  | Total average | 3.41 | 3.64 | 5.20 |
|  | Space and Furnishings | 3.50 | 3.62 | 4.71 |
|  | Personal Care Routines | 4.30 | 3.36 | 4.84 |
|  | Language and Literacy | 3.36 | 3.54 | 5.24 |
|  | Learning Activities | 2.21 | 2.96 | 4.42 |
|  | Interaction | 4.76 | 4.41 | 6.10 |
|  | Program Structure | 4.01 | 3.98 | 5.87 |
| Note. All data from 2017 to 2019 were included in the present study. Scores of Early et al. were calculated by the authors from Table 3 in Early, et al. [38]. Scores for the Montes et al. study were calculated by the authors using Table 1 in Montes, et al. [39]. | | | | |

| **Table 7.** Comparison of the ECERS-3 item scores among the three studies | | | |  |  |  |  |  |  |  |  |  |  |
| --- | --- | --- | --- | --- | --- | --- | --- | --- | --- | --- | --- | --- | --- |
|  | (1) Present study | | | (2) Early et al. | | | (3) Montes et al. | | | Test of differences (t-value) | |  |  |
|  | N | Mean | SD | N | Mean | SD | N | Mean | SD | (1) - (2) | (1) - (3) |  |  |
| Space and Furnishings |  |  |  |  |  |  |  |  |  |  |  |  |  |
| 1. Indoor space | 88 | 6.03 | 1.06 | 1063 | 4.55 | 1.55 | 148 | 5.70 | 1.47 | 1.48 | (8.81) | 0.33 | (1.87) |
| 2. Furnishings for care, play, and learning | 88 | 2.45 | 1.54 | 1063 | 4.05 | 1.10 | 148 | 4.97 | 1.28 | -1.60 | (-12.62) | -2.52 | (-13.52) |
| 3. Room arrangement for play and learning | 88 | 2.63 | 1.20 | 1063 | 3.42 | 1.45 | 148 | 4.47 | 1.83 | -0.80 | (-5.00) | -1.85 | (-8.44) |
| 4. Space for privacy | 88 | 2.75 | 1.27 | 1063 | 4.07 | 1.60 | 148 | 5.32 | 1.77 | -1.32 | (-7.54) | -2.57 | (-11.92) |
| 5. Child-related display | 88 | 2.64 | 1.80 | 1063 | 3.24 | 1.37 | 148 | 4.84 | 1.88 | -0.60 | (-3.87) | -2.20 | (-8.84) |
| 6. Space for gross motor play | 88 | 4.40 | 1.98 | 1063 | 3.18 | 1.42 | 148 | 3.68 | 1.73 | 1.22 | (7.47) | 0.72 | (2.92) |
| 7. Gross motor equipment | 88 | 3.64 | 2.16 | 1063 | 2.80 | 1.68 | 148 | 4.02 | 2.27 | 0.84 | (4.38) | -0.38 | (-1.28) |
| Personal Care Routines |  |  |  |  |  |  |  |  |  |  |  |  |  |
| 8. Meals/Snacks | 88 | 4.42 | 1.46 | 1063 | 3.15 | 1.29 | 148 | 4.70 | 1.79 | 1.27 | (8.78) | -0.28 | (-1.24) |
| 9. Toileting/diapering | 88 | 4.32 | 2.07 | 1063 | 3.21 | 1.41 | 148 | 4.42 | 2.11 | 1.11 | (6.79) | -0.10 | (-0.36) |
| 10. Health practices | 88 | 4.10 | 1.90 | 1063 | 3.06 | 1.40 | 148 | 4.75 | 1.99 | 1.04 | (6.51) | -0.65 | (-2.46) |
| 11. Safety practices | 88 | 4.38 | 2.48 | 1063 | 4.03 | 1.72 | 148 | 5.48 | 1.85 | 0.35 | (1.74) | -1.11 | (-3.90) |
| Language and Literacy |  |  |  |  |  |  |  |  |  |  |  |  |  |
| 12. Helping children expanding vocabulary | 88 | 3.05 | 1.41 | 1063 | 3.24 | 1.42 | 148 | 5.49 | 1.66 | -0.19 | (-1.24) | -2.44 | (-11.55) |
| 13. Encouraging children to use language | 88 | 3.80 | 1.65 | 1063 | 4.20 | 1.54 | 148 | 6.07 | 1.51 | -0.40 | (-2.36) | -2.27 | (-10.81) |
| 14. Staff use of books with children | 88 | 3.63 | 1.84 | 1063 | 3.38 | 1.69 | 148 | 4.97 | 1.91 | 0.25 | (1.30) | -1.35 | (-5.30) |
| 15. Encouraging children to use books | 88 | 3.48 | 1.20 | 1063 | 3.69 | 1.47 | 148 | 4.89 | 1.55 | -0.21 | (-1.32) | -1.41 | (-7.34) |
| 16. Becoming familiar with print | 88 | 2.86 | 1.37 | 1063 | 3.19 | 1.24 | 148 | 4.76 | 1.49 | -0.33 | (-2.35) | -1.90 | (-9.75) |
| Learning Activities |  |  |  |  |  |  |  |  |  |  |  |  |  |
| 17. Fine motor | 88 | 3.11 | 1.70 | 1063 | 3.98 | 1.59 | 148 | 5.55 | 1.59 | -0.87 | (-4.88) | -2.44 | (-11.08) |
| 18. Art | 88 | 2.95 | 1.78 | 1063 | 3.43 | 1.48 | 148 | 5.09 | 1.67 | -0.48 | (-2.85) | -2.14 | (-9.27) |
| 19. Music and movement | 88 | 2.35 | 1.25 | 1063 | 3.15 | 1.17 | 148 | 4.43 | 1.42 | -0.80 | (-6.11) | -2.08 | (-11.35) |
| 20. Blocks | 88 | 1.47 | 0.83 | 1063 | 2.23 | 1.26 | 148 | 3.59 | 1.51 | -0.76 | (-5.59) | -2.12 | (-12.15) |
| 21. Dramatic play | 88 | 2.16 | 1.39 | 1063 | 3.14 | 1.66 | 148 | 4.69 | 1.93 | -0.98 | (-5.39) | -2.53 | (-10.76) |
| 22. Nature/science | 88 | 2.35 | 1.37 | 1063 | 2.54 | 1.17 | 148 | 4.07 | 1.72 | -0.19 | (-1.43) | -1.72 | (-7.98) |
| 23. Math materials and activities | 88 | 1.41 | 0.89 | 1063 | 2.29 | 1.34 | 148 | 4.11 | 1.85 | -0.88 | (-6.06) | -2.70 | (-12.84) |
| 24. Math in daily events | 88 | 3.14 | 1.53 | 1063 | 2.99 | 1.43 | 148 | 4.81 | 1.76 | 0.15 | (0.92) | -1.67 | (-7.41) |
| 25. Understanding written numbers | 88 | 1.44 | 0.77 | 1063 | 1.73 | 1.15 | 148 | 3.01 | 2.05 | -0.29 | (-2.30) | -1.57 | (-6.89) |
| 26. Promoting acceptance of diversity | 88 | 1.70 | 0.75 | 1063 | 4.07 | 1.19 | 148 | 4.82 | 1.34 | -2.37 | (-18.35) | -3.12 | (-20.04) |
| 27. Appropriate use of technology | - | - | - | 291 | 3.14 | 1.86 | - | - | - | - | - | - | - |
| Interaction |  |  |  |  |  |  |  |  |  |  |  |  |  |
| 28. Supervision of gross motor | 84 | 5.08 | 1.76 | 1063 | 4.11 | 1.74 | 148 | 5.47 | 2.16 | 0.97 | (4.93) | -0.39 | (-1.40) |
| 29. Individualized teaching and learning | 88 | 3.95 | 1.78 | 1063 | 4.32 | 1.70 | 148 | 6.36 | 1.45 | -0.37 | (-1.93) | -2.41 | (-11.30) |
| 30. Staff-child interaction | 88 | 5.24 | 1.68 | 1063 | 4.97 | 1.84 | 148 | 6.47 | 1.27 | 0.27 | (1.32) | -1.23 | (-6.36) |
| 31. Peer interaction | 88 | 4.75 | 1.54 | 1063 | 4.47 | 1.56 | 148 | 6.12 | 1.28 | 0.28 | (1.62) | -1.37 | (-7.36) |
| 32. Discipline | 87 | 4.82 | 1.60 | 1063 | 4.18 | 1.42 | 148 | 6.08 | 1.54 | 0.64 | (3.98) | -1.26 | (-5.98) |
| Program Structure |  |  |  |  |  |  |  |  |  |  |  |  |  |
| 33. Transitions and waiting times | 88 | 4.3 | 1.76 | 1063 | 3.90 | 1.92 | 148 | 5.76 | 2.00 | 0.40 | (1.87) | -1.46 | (-5.68) |
| 34. Free play | 88 | 3.73 | 1.75 | 1063 | 4.06 | 1.51 | 148 | 5.98 | 1.5 | -0.33 | (-1.96) | -2.25 | (-10.48) |
| 35. Whole-group activities for play and learning | 76 | 4.84 | 1.77 | 1044 | 3.80 | 1.50 | - | - | - | - | - | - | - |
| Note. Data for the present study includes all data from 2017 to 2019. Scores of Early et al. were calculated by the authors using Table 3 from Early et al. [38]. Scores of Montes et al. were calculated by the authors using Table 1 from Montes et al. [39]. | | | | | | | | | | | |  |  |

| **Table 8.** Results of ANOVA for testing the score variations between nursery centers | | | | | |  |  |  |
| --- | --- | --- | --- | --- | --- | --- | --- | --- |
|  |  | Total average | Subscale 1 | Subscale 2 | Subscale 3 | Subscale 4 | Subscale 5 | Subscale 6 |
|  | %Explained | 43.40% | 42.85% | 35.65% | 37.38% | 44.98% | 31.96% | 43.03% |
|  | F-value | 3.70*** | 4.55*** | 2.14** | 2.32*** | 3.59*** | 1.84** | 3.33*** |
|  | BP-test (chi2) | 0.44 | 0.62 | 1.95 | 3.53* | 1.01 | 12.15*** | 0.06 |
| Note. N=88. Definitions of subscales. Subscale 1: Space and Furnishing. Subscale 2: Personal Care and Routines. Subscale 3: Language and Literacy. Subscale 4: Learning Activities. Subscale 5: Interaction. Subscale 6: Program Structure. In the calculation of the score for “Learning Activities”, the item “Appropriate use of technology” (item 27) was not included. In the calculation of “Program Structure”, item 35 “Whole-group activities for play and learning” was not included. The variations between facilities and between survey years were taken into account in ANOVA. %Explained: percentage of the total variation explained by the variation between facilities. BP-test (chi2): test statistics of Breusch-Pagan test. * : *p < .*10. ** : *p < .*05. *** : *p < .*01. | | | | | | | | |

| **Supplement information: Details of summary statistics of ECERS-3** | | | | | | | |
| --- | --- | --- | --- | --- | --- | --- | --- |
|  | Mean | SD | p10 | p25 | p50 | p75 | p90 |
| Total average | 3.41 | 0.69 | 2.52 | 3.06 | 3.39 | 3.80 | 4.21 |
| Space and Furnishings | 3.50 | 0.86 | 2.57 | 2.93 | 3.43 | 4.00 | 4.43 |
| Personal Care Routines | 4.30 | 1.21 | 2.75 | 3.50 | 4.25 | 5.00 | 6.00 |
| Language and Literacy | 3.36 | 0.92 | 2.20 | 2.70 | 3.20 | 3.80 | 4.80 |
| Learning Activities | 2.21 | 0.68 | 1.40 | 1.80 | 2.10 | 2.50 | 3.20 |
| Interaction | 4.76 | 1.24 | 3.00 | 4.00 | 5.00 | 5.60 | 6.20 |
| Program Structure | 4.01 | 1.44 | 2.00 | 3.00 | 4.00 | 5.00 | 6.00 |
| Note. All data from 2017 to 2019 were included in the present study. N=88. SD: standard deviation. p10, p25, p50, p75, p90: 10, 25, 50, 75, 90 percentiles. | | | | | | | |
